# Serum 25-hydroxyvitamin D levels among women newly diagnosed with breast cancer at Tikur Anbessa Specialized Hospital, Addis Ababa, Ethiopia

**DOI:** 10.1186/s12905-026-04453-8

**Published:** 2026-04-11

**Authors:** Mehari Meles, Gadissa Gutema, Melaku Tsegaye, Chala Bashea, Feyissa Challa, Demiraw Bikila, Gebremedhin Gebremicael, Tigst Getahun, Wossene Habtu, Mekdes Alem, Abebe Edao, Anna Carobene, Habteyes Hailu Tola

**Affiliations:** 1 Ethiopian Public Health Institute, Addis Ababa, Ethiopia; 2 College of Health Sciences, Addis Ababa University, Addis Ababa, Ethiopia; 3 College of Health Sciences, Wolaita Sodo University, Wolaita Sodo, Ethiopia; 4 Laboratory Medicine, IRCCS San Raffaele Scientific Institute, Milan, Italy; 5 College of Health Sciences, Salale University, Fitche, Ethiopia

**Keywords:** Cancer, Breast Cancer, Vitamin D deficiency, 25-Hydroxyvitamin D

## Abstract

**Background:**

Breast cancer (BC) is among the most frequently diagnosed cancers in women, accounting for approximately one in ten new cancer cases annually. The relationship between vitamin D status and BC risk remains unclear; while some observational studies suggest an association, others show no link, and causality is unproven. However, data from sub-Saharan Africa, and specifically Ethiopia, are scarce. This study aimed to assess serum 25-hydroxyvitamin D [25(OH)D] levels in Ethiopian women newly diagnosed with BC compared to healthy controls.

**Methods:**

A comparative cross-sectional study was conducted from January to March 2024 at Tikur Anbessa Specialized Hospital, Addis Ababa. Sixty-nine women with newly diagnosed BC and sixty-nine healthy controls were enrolled. Serum 25(OH)D concentrations were measured via electrochemiluminescence immunoassay. Data analysis was performed with SPSS v20. Median differences were evaluated using the Mann–Whitney U test with Hodges–Lehmann estimates and 95% confidence intervals (CI). Logistic regression identified factors associated with BC, and sensitivity analyses were conducted using alternative clinical cut-offs for severe vitamin D deficiency (SVDD).

**Results:**

Vitamin D deficiency was prevalent in both groups; however, SVDD (< 10 ng/mL) was significantly more prevalent among BC patients than among controls (26.1% vs. 7.2%, *p* = 0.006). A significant median difference in 25(OH) D levels was observed between cases and controls (median difference: 2.1 ng/mL; 95% CI: 0.07–4.4; *p* = 0.043). Serum 25(OH)D levels were also significantly lower in stage IV BC compared with controls and stage I. SVDD was independently associated with BC (adjusted odds ratio = 7.1; 95% CI: 1.7–30.1), and this association remained robust when alternative clinical cut-offs were applied. Reduced sunlight exposure and overweight/obesity were also independently associated with BC.

**Conclusion:**

SVDD and higher Body Mass Index were significantly associated with BC status. Lower 25(OH)D levels are most pronounced in metastatic (Stage IV) disease. While reduced sunlight exposure was also associated with BC, the direction of these relationships cannot be determined with certainty. These findings highlight an association that warrants further investigation. Further prospective studies with pre-diagnostic 25(OH)D measurements are needed to clarify causality and to determine whether strategies to address VDD may contribute to BC risk reduction.

## Background

Breast Cancer (BC) remains the most frequently diagnosed cancer among women worldwide and is a leading cause of cancer-related mortality. Although BC can occur in both sexes, it is approximately 100 times more common in women. Globally, it accounts for about one in ten new cancer diagnoses annually, highlighting its substantial public health burden [1, 2].

The development of BC is multifactorial, involving a combination of genetic, hormonal, environmental, and lifestyle-related risk factors. Established contributors include age, family history of BC, hormonal exposure, smoking, obesity, and reproductive history. Additionally, certain inherited genetic mutations, such as BRCA1 and BRCA2, are known to increase susceptibility [3, 4]. On the other hand, protective factors may include regular physical activity, a balanced diet high in fiber, and adequate vitamin D status [5].

Vitamin D is a fat-soluble vitamin primarily obtained through synthesis in the skin upon exposure to ultraviolet B (UVB) radiation from sunlight, with smaller amounts derived from dietary sources [6]. The two main forms are vitamin D2 (ergocalciferol) and vitamin D3 (cholecalciferol). The active form, 1,25-dihydroxyvitamin D3 [1,25(OH)2D3], regulates calcium and phosphate balance in the body. Its biological effects are mediated by binding to the vitamin D receptor (VDR), a transcription factor expressed in various tissues, including the intestine, kidney, bone, and several normal and malignant tissues. Through these actions, vitamin D influences cell proliferation, differentiation, and immune function [7–9]. These biological roles have led to increasing interest in the potential impact of vitamin D deficiency (VDD) on cancer development and progression [10]. The serum concentration of 25-hydroxyvitamin D [25(OH)D], which reflects the combined levels of vitamin D2 and D3, is widely used as the best measure of vitamin D status in clinical and research settings [11, 12].

VDD is a significant global public health issue affecting a substantial proportion of the population worldwide [13], and associated with numerous health consequences, including an increased risk of BC [14]. A prior systematic review found that 30–90% of people in developing nations suffered from VDD, though cut-off values varied by region [15]. Despite abundant sunlight, VDD is particularly prevalent in many African countries due to factors such as higher skin melanin content (which reduces cutaneous vitamin D synthesis), clothing practices that limit UVB exposure, and limited dietary vitamin D intake [16, 17]. A systematic analysis of 19 African countries, including Ethiopia, reported that approximately 30% of individuals were VDD, and 20% experienced severe vitamin D deficiency (SVDD) [18].

Observational studies have frequently reported an association between low 25(OH)D status and increased BC risk, with the prevalence of VDD among women with BC ranging from 23% to 95.6% across different populations [19]. For example, a study from Indonesia found that 82.4% of BC patients had SVDD, defined as serum 25(OH)D levels below 12 ng/mL [20]. Similarly, more than half of newly diagnosed premenopausal women with BC in Iran were reported to have very severe or SVDD [21]. A multiethnic cohort study among postmenopausal women showed that those with deficient 25(OH)D levels had a 7.5-fold higher risk of BC compared with controls [22], while an integrative review suggested that serum 25(OH)D concentrations ≥ 40.6 ng/mL may be protective from BC [23]. In Ethiopia, 86% of BC patients were VDD, with 41.1% having SVDD, defined as < 10 ng/mL [24]. However, not all studies are consistent, and several large cohort studies have reported no clear association, underscoring ongoing uncertainty in this field [25, 26].

Because vitamin D is essential for calcium and phosphate metabolism, assessing these minerals alongside vitamin D provides a more comprehensive understanding of metabolic changes in breast cancer. Disruptions in calcium-phosphate balance have been observed in several malignancies and may indicate cancer-related alterations in bone and mineral homeostasis [27–29]. In this study, we primarily defined SVDD as circulating 25(OH)D levels ≤ 10 ng/mL, VDD as > 10 and < 20 ng/mL, and levels ≥ 20 ng/mL as sufficient [24, 30], consistent with the Endocrine Society’s clinical practice guideline, which defines deficiency as < 20 ng/mL [31]. However, definitions of optimal vitamin D status, particularly for non-skeletal outcomes, remain debated, and alternative thresholds (such as ≤ 12 ng/mL for SVDD) are also used in the literature [32, 33]. Therefore, we performed a sensitivity analysis using this alternative definition.

Few studies have evaluated these biochemical markers alongside vitamin D in Ethiopian breast cancer patients or, more broadly, in African populations. Despite growing global research, data from sub-Saharan Africa, and Ethiopia in particular are scarce. To address this knowledge gap, this study assessed serum 25(OH)D levels and their association with breast cancer among Ethiopian women. Therefore, this study aimed to assess serum 25(OH)D levels and their association with BC among Ethiopian women, addressing a critical knowledge gap in this population.

## Materials and methods

### Study design and area

A comparative cross-sectional study was conducted from January to March 2024 at the adult oncology unit of Tikur Anbessa Specialized Hospital (TASH) in Addis Ababa, Ethiopia to assess serum 25(OH) D levels, and their association with BC among Ethiopian women. This hospital is the country’s largest referral center and provides comprehensive oncologic and diagnostic services. It was purposefully selected due to its high volume of newly diagnosed BC cases [34].

### Inclusion and exclusion criteria

The study population consisted of women with newly diagnosed, histopathological confirmed breast cancer, irrespective of menopausal status. The control group included women without a history of cancer who attended the same hospital as outpatients for routine medical examinations or for minor, non-chronic conditions such as mild upper respiratory infections, minor gastrointestinal complaints, non-chronic musculoskeletal pain, or preventive health visits. Individuals with chronic diseases, severe illness, pregnancy, or current vitamin D supplementation were excluded to minimize potential confounding of serum 25(OH)D, calcium, and phosphate levels.

### Sample size calculation and sampling technique

A total of 138 participants were enrolled, with 69 in each group. Cases were selected consecutively as they presented for initial treatment. Controls were selected from the same hospital, provided they met the inclusion criteria and had no history of BC. Eligible individuals were listed and randomly chosen using a computer-generated sequence.

### Data collection and quality assurance

Structured interviews and medical chart reviews were used to gather socio-demographic and clinical information. We collected data using a structured questionnaire that was originally written in English and then translated into Amharic for the participants. The principal investigator designed the questionnaire using relevant literature and the study’s objectives. The full English version can be found in the first author’s master’s thesis, which is available in the Addis Ababa University Institutional Repository [35]. BC stage (I-IV) and histological type data were taken directly from patient medical records as documented by the hospital pathology and oncology departments. We used calibrated digital equipment to assess body weight and height, and calculated Body Mass Index (BMI) as weight in kilograms divided by height in meters squared. Participants were classified as underweight/normal (BMI < 25 kg/m²) or overweight/obese (BMI ≥ 25 kg/m²). Sunlight exposure was self-reported and categorized as either less than 30 min per day or 30 min or more per day. To validate the questionnaire, a pilot test of 10 cases and 10 controls was carried out. Data collection was carried out by trained individuals under supervision, and quality checks were undertaken on a regular basis to guarantee accuracy and consistency. 

### Laboratory tests

Venous blood samples (3–5 mL) were taken from each participant at TASH by trained nurses using serum separator tubes and conventional aseptic methods. Within 30 min after collection, samples were allowed to clot before being centrifuged for five minutes at 4000 revolutions per minute. The serum was then aliquoted into labeled 1.5 mL nunc tubes and sent in temperature-controlled containers to the EPHI’s National Clinical Chemistry Reference Laboratory (NCCRL). Serum samples were kept at −70 °C for up to 3–4 months before analysis. To ensure stability and avoid degradation by multiple freeze-thaw cycles, all samples were thawed once during measurement.

25(OH)D, Calcium, and inorganic phosphate were measured on a Cobas 6000 analyzer (Roche Diagnostics). 25(OH)D was quantified by electrochemiluminescence immunoassay (Elecsys Vitamin D total III; lot 78688703), calibrated with VITD3 Cal 1/2 (lot 77381302), and controlled using PreciControl Vitamin D (lots 74642399/499). Ca (CA2 Gen.2; lot 794470) and phosphate (PHOS2 ver.2; lot 801953) were determined photometrically, calibrated with Calibrator for All Systems (CFAS; lot 72135500), and monitored with PreciControl ClinChem Multi 1 & 2 (lots 7049‑6600/9100).

According to the manufacturer’s inserts, the intermediate (inter-assay) precision for serum on this platform is as follows: 25(OH)D, ≤ 9.8%; Calcium, ≤ 2.5%; and phosphate, ≤ 1.4%. The 25(OH)D assay is traceable to the ID-LC-MS/MS Reference Measurement Procedure and National Institute of Standards and Technology Standard Reference Material (NIST SRM) 2972. The Ca assay is traceable to NIST SRM 956c, while the phosphate assay uses a NIST-traceable primary reference material [36–38]. All measurements were performed in an ISO 15,189-accredited laboratory (Ethiopian Accreditation Service) that participates in an External Quality Assessment Scheme.

### Vitamin D status categorization

Serum 25(OH)D concentrations were categorized for analytical purposes. The primary analysis classified participants into SVDD (≤ 10.0 ng/mL), VDD (10.1–19.9 ng/mL), and sufficient levels (≥ 20.0 ng/mL). To assess the robustness of this classification, a sensitivity analysis was performed using a more inclusive threshold for SVDD (≤ 12.0 ng/mL), with VDD defined as 12.1–19.9 ng/mL. All 25(OH) D values are reported in ng/mL, the unit used by the clinical laboratory and most relevant to the local clinical context. For reference, the approximate conversion to SI units is 1 ng/mL ≈ 2.5 nmol/L.

### Data analysis

Data entry was performed in Microsoft Excel, followed by accuracy cross-checking and analysis using SPSS version 20. Descriptive statistics were used to summarize demographic and clinical characteristics. The Shapiro–Wilk test was applied to assess normality; non-normally distributed continuous variables are reported as medians with interquartile ranges (IQR). Group comparisons utilized the Mann-Whitney U test for two groups and the Kruskal-Wallis test for more than two groups, with post-hoc testing as appropriate. Median differences and 95% confidence intervals were estimated using the Hodges–Lehmann method. Trends across ordered BC stages were evaluated using the Jonckheere–Terpstra test. Logistic regression identified factors associated with BC. Model fit was assessed using the Hosmer–Lemeshow test, multicollinearity was evaluated using variance inflation factors (VIF < 2.5), and discrimination was measured by the area under the ROC curve (AUC). Statistical significance was defined as *p* < 0.05. The primary outcome was the pre-specified comparison of median serum 25(OH)D levels between BC patients and healthy controls. All other analyses were considered secondary or exploratory and are reported without adjustment for multiple testing.

## Results

### Baseline characteristics of study participants

The sociodemographic, lifestyle, and clinical characteristics of the 69 BC patients and 69 matched controls are presented in Table 1. BC patients had significantly higher prevalence of overweight/obesity (31.9% vs. 10.1%, *p* = 0.002) and lower daily sunlight exposure (34.8% vs. 13.0% with < 30 min/day, *p* = 0.003) compared to controls. No other variables showed statistically significant differences between groups. 

**Table 1:** Sociodemographic and clinical characteristics of BC patients and controls

Variables	Classifications	Cases (*n* = 69)	Controls (*n* = 69)	*P*-Value
		Frequency (%)	Frequency (%)	
Age group	< 50	44 (63.8)	48 (69.6)	0.470
	*≥* 50	25 (36.2)	21 (30.4)	
Marital status	Single	15 (21.7)	13 (18.8)	0.672
	Married	54 (78.3)	56 (81.2)	
Educational	No formal education	29 (42.1)	26 (37.7)	
Status	Secondary or less	23 (33.3)	35 (50.7)	0.053
	Diploma and above	17 (24.6)	8 (11.6)	
Occupation	Unemployed	6 (8.7)	9 (13.0)	0.412
	Employed	63 (91.3)	60 (87.0)	
BMI	< 25 kg/m^2^	47 (68.1%)	62 (89.9)	**0.002**
	≥ 25 kg/m^2^	22 (31.9)	7 (10.1)	
Parity Number	None (0)	9(13.0)	11 (15.9)	
	1–3	33 (47.8)	32 (46.4)	0.889
	**≥** 4	27 (39.1)	26 (37.7)	
Menopause	Pre menopause	28 (40.6)	38 (55.1)	0.088
	Post menopause	41 (59.4)	31 (44.9)	
Smoking	Yes	10 (14.5)	4 (5.8)	0.091
Condition	No	59 (85.5)	65 (94.2)	
Contraceptive	Yes	32 (46.4)	22 (31.9)	0.485
	No	37 (53.6)	47 (68.1)	
Exposed to	< 30 min/day	24 (34.8)	9 (13.0)	**0.003**
Sunlight	**≥** 30 min/day	45 (65.2)	60 (87.0)	

Data are presented as frequency (percentage). P-values were calculated using Chi-square test. BMI = Body Mass Index. Bold values denote statistical significance (*p* < 0.05)

### Correlation matrix for 25(OH)D with BMI, age, and sunlight exposure

Bivariate Spearman correlations revealed no significant associations between serum 25(OH)D and age, BMI, or categorized sunlight exposure (all *p* > 0.05). Age showed a weak positive correlation with phosphate (*r* = 0.242, *p* = 0.004) (Table 2).

**Table 2:** Correlation matrix for 25(OH)D with BMI, age, and sunlight exposure

Variable	25(OH)D	Age	BMI	Calcium	Phosphate	Sun-Exposure†
25(OH)D	1.0					
Age	−0.061	1.0				
BMI	−0.015	0.107	1.0			
Calcium	0.145	0.043	−0.078	1.0		
Phosphate	0.121	**0.242**	−0.166	0.037	1.0	
Sun Exposure†	−0.013	−0.083	0.050	0.099	−0.097	1.0

Bold indicates *p* < 0.01. †Sun exposure coded as 0 (< 30 min/day) or 1 (≥ 30 min/day)

### Biochemical parameters among the stages of BC patients

A Jonckheere-Terpstra trend test was conducted to assess whether 25(OH)D levels decrease with increasing disease severity across BC stages (I to IV). Although the analysis showed a consistent downward trend (Std. J-T Statistic = −1.9), it did not reach statistical significance (*p* = 0.061). Notably, only stage IV patients had significantly lower 25(OH)D levels compared to both the control group and stage I (Table 3).

**Table 3:** Biochemical parameters in controls and in the BC stage

Parameters	Controls(*n* = 69)	Stage I (*n* = 11)	Stage II (*n* = 25)	Stage III (*n* = 19)	Stage IV (*n* = 14)	*P*-Value
25(OH)D	14.8(12.7–19.7) ^ac^	16.2(14.5–22.5) ^bc^	12.1(9–15.3)	15.3(10.4–19.1)	9.5(6.5–16.1)^c^	**< 0.05** ^abc^
Ca	2.3(2.1–2.4)	2.3(2.3–2.4)	2.2(2.1–2.4)	2.3(2.2–2.4)	2.1(1.7–2.3)	0.073
Phosphate	1.1(0.94–1.3)	1.0(0.87–1.1)	1.1(0.92–1.2)	1.2(1.0–1.3)	1.1(0.91–1.5)	0.061

25(OH)D:25-hydroxyvitamin D in ng/mL; total calcium in mmol/L, and phosphate in mmol/L. Values were presented as median (IQR); IQR = interquartile Range. Superscript letters (a, b, c) indicate results of the Kruskal–Wallis test followed by post-hoc pairwise comparisons: Superscript ‘bc’ indicates a significant difference between stage I and IV; similarly, ‘ac’ indicates a significant difference between control and stage IV (*p* < 0.05). A Jonckheere-Terpstra test for an ordered trend across stages I–IV showed a non-significant decreasing trend in 25(OH)D levels (J-T statistic = −1.9, *p* = 0.061)

A sensitivity analysis that excluded Stage IV patients was conducted to determine whether the observed vitamin D deficit was specific to advanced disease. Comparison of non-metastatic BC cases (Stages I–III combined) with controls revealed no statistically significant difference in 25(OH)D levels (Mann-Whitney U test = 1662.5; *p* = 0.237). Analysis of non-metastatic stages individually (Stages I, II, III, and controls) using the Kruskal-Wallis test, none of the pairwise comparisons were statistically significant (*p* > 0.05).

### Comparison of serum biochemical parameters between BC case and control groups

**Fig. 1:**
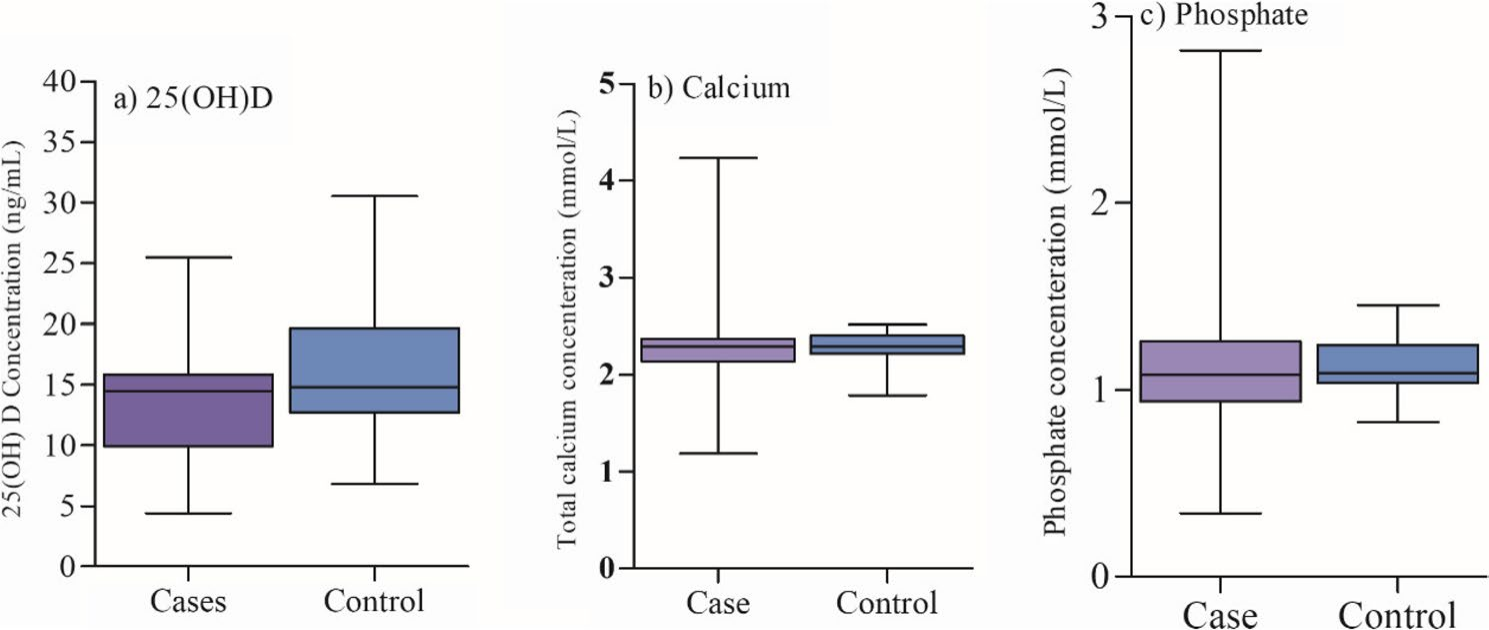
Median serum concentrations of (**a**) 25-hydroxyvitamin D [25(OH)D] (ng/mL), (**b**) total calcium (mmol/L), and (**c**) phosphate (mmol/L) in women with BC (*n* = 69) and controls (*n* = 69). Boxplots represent the median and interquartile range (IQR). Exact p-values from the Mann–Whitney U test are shown: 25(OH)D: *p* = 0.043, Calcium: *p* = 0.162, Phosphate: *p* = 0.267

Figure 1 shows the comparison of serum 25(OH)D, calcium, and phosphate levels between cases and controls. The median (IQR) serum 25(OH)D concentration was 14.5 (9.9–15.8) ng/mL in cases and 14.8 (12.7–19.7) ng/mL in controls. The Mann–Whitney U test showed a statistically significant median difference (Hodges–Lehmann median difference: 2.1 ng/mL, 95% CI: 0.07 to 4.4; *p* = 0.043). There was no significant median difference in serum calcium between cases (median = 2.3 mmol/L, IQR = 2.1–2.4 mmol/L) and controls (median = 2.3 mmol/L, IQR = 2.2–2.4 mmol/L) (median difference: 0.03 mmol/L, 95% CI: −0.02 to 0.09; *p* = 0.162). Similarly, there was no significant median difference in serum phosphate levels between cases (median = 1.1 mmol/L, IQR = 0.94–1.3 mmol/L) and controls (median = 1.1 mmol/L, IQR = 1.0–1.2 mmol/L) (median difference: 0.03 mmol/L, 95% CI: −0.03 to 0.10; *p* = 0.267).

### Vitamin D status with different clinical definitions in BC patients and controls

To assess the dependence of the findings on the definition of SVDD, results were compared using two common clinical cutoffs. The table below demonstrates that, whether SVDD is defined as levels at or below 10 ng/mL or at or below 12 ng/mL, it remains significantly more prevalent in BC patients than in healthy controls. The association between SVDD and BC was robust and statistically significant for both definitions (Table 4).

**Table 4:** Vitamin D status prevalence with alternative SVDD cutoffs (≤ 10 ng/mL vs. ≤12 ng/mL)

Group	Deficiency Category	*≤* 10 ng/mL cutoff	*≤* 12 ng/mL cutoff
		Frequency (%)	Frequency (%)
Cases	SVDD	18(26.1)	27(39.1)
	VDD	41(59.4)	32(46.4)
	Sufficient	10(14.5)	10(14.5)
Controls	SVDD	5(7.2)	13(18.8)
	VDD	45(65.2)	37(53.6)
	Sufficient	19(27.5)	19(27.5)
Association (χ²)		*p* = 0.006	*p* = 0.018

SVDD (Severe Vitamin D Deficient) defined as ≤ 10 ng/mL or ≤ 12 ng/mL. VDD (Vitamin D Deficient) ranges between: >10 and < 20 ng/mL cutoff 10 ng/mL; >12 and < 20 ng/mL cutoff 12 ng/mL. Sufficient as levels of 20.0 ng/mL or higher for both. p-values from Pearson’s chi‑square test for association between vitamin D status (Sufficient/VDD/SVDD) and case/control group

### Clinical, sociodemographic, and vitamin D status factors associated with BC

Bivariate analysis showed associations between BC and limited sun exposure, higher BMI, lower education level, and SVDD. Variables of clinical importance, along with those with *p* < 0.20 in the bivariate analysis, were entered into the multivariable model. Age, parity, and contraceptive use were retained in the adjusted analysis due to their established relevance to BC. After controlling for potential confounders, individuals with SVDD had 7.1 times higher odds of having BC compared to those with sufficient vitamin D. Reduced sunlight exposure (< 30 min/day) and BMI(≥ 25 kg/m^2^) also remained significantly associated with BC in the adjusted model (Table 5).

**Table 5:** Multivariable analysis of factors associated with BC, including clinical, sociodemographic characteristics, and vitamin D status

Variable		COR (95%CI)	*P*-value	AOR (95%CI)	*P*-value
Menopausal	Pre-menopause	1		1	
Status	Post-menopause	1.8(0.91–3.5)	0.090	1.8(0.60–5.3)	0.293
Smoking	No	1		1	
	Yes	2.8(0.82–9.3)	0.101	4.1(0.92–18.1)	0.064
Sun-Exposure	*≥* 30 min/day	1		1	
	< 30 min/day	3.6(1.5–8.4)	**0.004**	3.9(1.4–10.6)	**0.008**
BMI	< 25 kg/m^2^	1		1	
	*≥* 25 kg/m^2^	4.2(1.6–10.5)	**0.003**	5.0(1.6–15.2)	**0.005**
Educational	Diploma and above	1		1	
Status	Secondary or less	0.31(0.12–0.83)	**0.020**	0.29(0.09–0.92)	0.068
	Illiterate	0.53(0.19–1.4)	0.203	0.50(0.15–1.7)	0.263
	Sufficient	1		1	
Vitamin D	SVDD	6.8(1.9–23.9)	**0.003**	7.1(1.7–30.1)	**0.008**
Status	VDD	1.7(0.72–4.2)	0.219	2.3(0.86–6.4)	0.099
Age	< 50 Years	1		1	
	*≥* 50 Years	1.3(0.64–2.6)	0.471	0.43(0.13–1.4)	0.166
Contraceptive	No	1		1	
Use	Yes	1.3(0.64–2.5)	0.486	1.7(0.74–4.0)	0.212
Parity	No child	1		1	
Number	1–3	0.79(0.28–2.2)	0.651	0.51(0.14–1.9)	0.308
	≥ 4	1.0(0.48–2.1)	0.985	0.89(0.36–2.2)	0.811

AOR, adjusted odds ratio; CI, confidence interval; SVDD (Severe Vitamin D Deficient) defined as ≤ 10 ng/mL ng/mL. VDD (Vitamin D Deficient) ranges: >10 and < 20 ng/mL and Sufficient ≥ 20 ng/mL; BMI, body mass index; Ref, reference group; 1, reference category; COR, crude odds ratio. Odds ratios are adjusted for all other variables listed in the table. An AOR greater than 1 indicates increased odds of BC, while an AOR less than 1 indicates decreased odds. Bold values denote statistical significance (*p* < 0.05). The model demonstrated acceptable fit (Hosmer-Lemeshow *p* = 0.421) and discrimination (AUC = 0.786)

In a sensitivity analysis using an alternative clinical cutoff for SVDD (*≤* 12 ng/mL instead of *≤* 10 ng/mL), the association persisted, with SVDD associated with five-fold higher odds of BC (AOR = 5.0, 95% CI: 1.5–16.3, *p* = 0.007).When vitamin D status was treated as an ordinal variable (Sufficient = 1, VDD = 2, SVDD = 3), a significant linear trend was observed: each worsening deficiency category was associated with 2.5 times higher odds of BC (95% CI: 1.4–4.5, *p* = 0.003).

## Discussion

This comparative study conducted at TASH found that women newly diagnosed with breast cancer had significantly lower serum 25(OH)D concentrations than controls. The prevalence of SVDD (≤ 10 ng/mL) was markedly higher in cases (26.1%) compared to controls (7.2%). It was significantly associated with breast cancer status after multivariable adjustment (AOR = 7.1, 95% CI: 1.7–30.1). By contrast, total calcium and inorganic phosphate did not differ materially between groups. Notably, low 25(OH)D levels were most pronounced in Stage IV disease, with concentrations significantly lower than those of both controls and Stage I patients. These findings demonstrate an association between low vitamin D status and BC at diagnosis, although causality cannot be inferred due to the cross-sectional design.

Sensitivity analyses supported the robust association between BC status and VDD. Applying a conventional clinical cutoff for SVDD (≤ 12 ng/mL), the prevalence of SVDD remained notably higher among cases (39.1%) compared to controls (18.8%), and the association remained significant (AOR = 5.0, 95% CI: 1.6–25.8). A trend toward decreasing 25(OH)D with advancing cancer stage was also observed, though it did not reach statistical significance (*p* = 0.062). This pattern is consistent with some clinical observations that SVDD may be more common in metastatic disease [39].

Our findings are broadly consistent with previous studies reporting lower vitamin D in BC patients [24, 30]. However, some large cohort studies have reported null associations [40–42], including a recent 2024 analysis [25], This inconsistency suggests that the role of vitamin D in BC remains debated and is not yet established as a causal factor. This heterogeneity likely reflects differences in study design, population characteristics, sunlight exposure and measurement methods.

Adiposity is known to lower circulating 25(OH)D through tissue sequestration and volumetric dilution [43, 44], In our study, 31.9% of cases and 10.1% of controls had BMI ≥ 25 kg/m², consistent with previous evidence linking higher BMI to both lower vitamin D and increased BC risk [40, 45, 46]. Although we adjusted for BMI, residual confounding by body size remains possible. Bi-directional Mendelian randomization studies further suggest that higher BMI causally lowers 25(OH)D, rather than the reverse [47], reinforcing our conservative interpretation. In practical terms, both clinical dosing and statistical modeling should consider body size; where feasible, future analyses should incorporate non-linear BMI terms or direct adiposity measures (e.g. fat mass) to better reflect dilution effects.

Sunlight exposure, a key determinant of vitamin D synthesis, was also independently associated with breast cancer status in this study. Because only outdoor exposure enables cutaneous vitamin D production, we distinguished between overall physical activity and time spent outdoors [48]. Women reporting less than 30 min of daily sun exposure had higher odds of BC compared with those reporting longer exposure. Similar associations between lower sunlight exposure and increased breast cancer risk have been reported in other populations [49, 50]. However, sunlight exposure in our study was self-reported and did not account for clothing coverage, time of day, or skin phototype, all of which may influence cutaneous vitamin D synthesis. These factors should be considered when interpreting the observed association.

In addition to environmental factors, several population-specific determinants may contribute to VDD in African settings [51]. Higher melanin content, cultural clothing practices that limit skin exposure, and limited dietary fortification of vitamin D may all contribute to lower circulating 25(OH)D levels. These factors were not quantitatively measured in this study but represent important areas for future research.

Although cases and controls were enrolled concurrently (January–March), which helps reduce between-group seasonal bias, single-season sampling may limit the generalizability of absolute 25(OH)D concentrations because vitamin D levels fluctuate throughout the year [52, 53]. Future studies including multiple seasons would provide a more complete assessment of vitamin D status.

No material differences in total calcium or phosphate were observed between cases and controls. However, albumin and ionized calcium were not measured; therefore, subtle alterations in calcium homeostasis [54] may have been underestimated, and the findings regarding calcium status should be interpreted with caution.

From a clinical perspective, VDD may have implications beyond bone metabolism, including potential effects on immune modulation, inflammation, and cellular proliferation [7–10]. In oncology settings, low 25(OH)D levels have been associated in some studies with poorer prognosis, reduced treatment tolerance, and adverse outcomes, although evidence remains inconsistent [9, 14, 23]. In the present study, a high prevalence of deficiency, particularly among patients with advanced disease, was observed. However, given the cross-sectional design, it is not possible to determine whether low 25(OH)D status contributes to disease development or reflects disease-related factors such as reduced mobility, systemic illness, or decreased sunlight exposure. Therefore, while these findings highlight a potentially relevant association, they should be interpreted as hypothesis-generating and do not support routine screening or supplementation specifically for breast cancer management at this stage.

In populations where cultural or religious clothing practices limit sun exposure, VDD may be particularly common [55, 56]. However, the direct impact of vitamin D supplementation on cancer outcomes remains uncertain, and further research, particularly well-designed prospective studies, is needed to determine whether maintaining adequate vitamin D levels influences treatment tolerance, quality of life, or disease progression.

This study has several methodological strengths. We recruited newly diagnosed, treatment-naïve BC cases, minimizing the potential influence of cancer therapy on 25(OH)D levels. All biochemical analyses were performed in an ISO 15,189-accredited laboratory using standardized electrochemiluminescence immunoassays, with documented traceability and participation in external quality assessment programs. To enhance transparency and interpretability, we report effect sizes with 95% confidence intervals (including Hodges-Lehmann median differences for non-parametric comparisons) and provide model diagnostics for multivariable analyses, such as the area under the ROC curve and calibration statistics.

Several limitations should be considered when interpreting our findings.

Selection bias. The control group consisted of women attending the same hospital for routine check-ups or minor non-chronic conditions, such as mild respiratory infections or preventive visits. Although these conditions are unlikely to directly influence vitamin D metabolism, hospital-based controls may differ from the general population in health-seeking behavior or time spent indoors, which could influence sunlight exposure and vitamin D status. We also lacked data on shift work or inpatient status among controls, factors that may affect sunlight exposure.

Precision of estimates. The association between SVDD and BC should be interpreted cautiously. The estimated odds ratio was accompanied by a wide confidence interval (1.7–30.1), indicating limited precision. This imprecision likely reflects the modest sample size and the relatively small number of participants in the SVDD subgroup, which may affect model stability and suggests that the observed association should be considered exploratory.

Measurement of sunlight exposure. Sunlight exposure was self-reported and categorized into broad groups, which may have led to exposure misclassification. Additionally, important determinants of vitamin D synthesis, such as clothing coverage, and skin phototype, were not assessed.

Seasonality. Recruitment was limited to the months of January through March. Although cases and controls were enrolled concurrently to minimize seasonal bias, single-season sampling may limit the generalizability of the absolute 25(OH)D concentrations reported.

Study design. Finally, the cross-sectional design precludes any conclusions about causality. Reverse causation is possible, as BC symptoms or disease-related behavioral changes may have reduced outdoor activity and consequently lowered 25(OH)D levels prior to diagnosis.

## Conclusion

Women newly diagnosed with BC had significantly lower serum 25(OH)D levels and a higher prevalence of SVDD compared to controls. The lowest 25(OH)D concentrations were observed in stage IV disease. SVDD, reduced sunlight exposure, and higher BMI were all significantly associated with BC in this study.

These cross-sectional findings highlight an important association but do not establish causality due to potential confounding by factors such as adiposity, dietary habits, and the possibility of reverse causation (where advanced disease may itself lower 25(OH)D). To clarify the direction and clinical relevance of this relationship, prospective, season-spanning studies are warranted. Such studies should incorporate with: Pre-diagnostic vitamin D measurements, objective assessment of sunlight exposure, detailed diet supplement, and adiposity data, and LC–MS/MS-standardized 25(OH)D quantification. This research is needed to determine whether strategies to improve vitamin D status could influence BC risk or progression in this population.

## Data Availability

The datasets generated and/or analyzed during the current study are available from the corresponding author upon reasonable request.

## Abbreviations

BC: Breast Cancer
BMI: Body Mass Index
ECLIA: Electro Chemiluminescent immunoassay
EPHI: Ethiopian Public Health Institute
LC: MS/MS-Liquid Chromatography-Tandem Mass Spectrometry
NCCRL: National Clinical Chemistry Reference Laboratory
25(HO)D: 25 hydroxyvitamin D
NIST: National Institute of Standards and Technology
SRM: Standard Reference Material
SVDD: Severe Vitamin D Deficiency
WHO: World Health Organization
